# Detection of Glaucoma on Fundus Images Using Deep Learning on a New Image Set Obtained with a Smartphone and Handheld Ophthalmoscope

**DOI:** 10.3390/healthcare10122345

**Published:** 2022-11-22

**Authors:** Clerimar Paulo Bragança, José Manuel Torres, Christophe Pinto de Almeida Soares, Luciano Oliveira Macedo

**Affiliations:** 1 ISUS Unit, Faculdade de Ciência e Tecnologia, Universidade Fernando Pessoa, 4249-004 Porto, Portugal; 2 Artificial Intelligence and Computer Science Laboratory, LIACC, University of Porto, 4100-000 Porto, Portugal; 3 Department of Ophthalmology, Eye Hospital of Southern Minas Gerais State, R. Joaquim Rosa, 14, Itanhandu 37464-000, MG, Brazil

**Keywords:** smartphone ophthalmoscope, fundus image dataset, panoptic ophthalmoscope, glaucoma, ensemble CNN

## Abstract

Statistics show that an estimated 64 million people worldwide suffer from glaucoma. To aid in the detection of this disease, this paper presents a new public dataset containing eye fundus images that was developed for glaucoma pattern-recognition studies using deep learning (DL). The dataset, denoted Brazil Glaucoma, comprises 2000 images obtained from 1000 volunteers categorized into two groups: those with glaucoma (50%) and those without glaucoma (50%). All images were captured with a smartphone attached to a Welch Allyn panoptic direct ophthalmoscope. Further, a DL approach for the automatic detection of glaucoma was developed using the new dataset as input to a convolutional neural network ensemble model. The accuracy between positive and negative glaucoma detection, sensitivity, and specificity were calculated using five-fold cross-validation to train and refine the classification model. The results showed that the proposed method can identify glaucoma from eye fundus images with an accuracy of 90.0%. Thus, the combination of fundus images obtained using a smartphone attached to a portable panoptic ophthalmoscope and artificial intelligence algorithms yielded satisfactory results in the overall accuracy of glaucoma detection tests. Consequently, the proposed approach can contribute to the development of technologies aimed at massive population screening of the disease.

## 1. Introduction

In recent years, scientific efforts and technological advances have been applied to ophthalmic technology to provide quality eye care, which is an important factor in assessing the progression of eye diseases and excellence in treatment outcomes; however, this progress has not kept up with the ophthalmic care needs of the population. Estimates from the World Health Organization (WHO) point out that globally, at least 2.2 billion people have a visual impairment and, of these, at least 1 billion people have a visual impairment that could have been avoided or has not yet been treated. These statistical data may be related to the lack of consent of the severity of eye diseases by a part of the population or to the burden of eye diseases and visual impairment, which tends to penalize middle- or low-income countries and the poorest populations [1].

Statistics also indicate that the number of people suffering from eye disease, visual impairment, and blindness will increase in the coming decades due to population growth and aging, as well as behavioral and lifestyle changes and urbanization [1].

The importance of eye diseases that do not usually cause vision impairment should not be underestimated. However, eye diseases that can lead to visual impairment and blindness are naturally at the heart of prevention and intervention strategies. Among these diseases we can highlight age-related macular degeneration (ARMD), cataracts, diabetic retinopathy (DR), trachoma, and glaucoma. After trachoma and cataracts, glaucoma is the third leading cause of blindness worldwide;however, trachoma is a preventable disease, whereas cataracts are reversible, and glaucoma is the most important of these diseases considering that it can lead to irreversible blindness. Statistics show that an estimated 64 million people worldwide suffer from glaucoma, of which 6.9 million are reported to have only moderate or severe distance vision impairment or blindness due to more severe forms of the disease [1,2].

Glaucoma can affect the fundus of the eye and thereby cause gradual loss of vision and, in severe cases, blindness. This condition is characterized by changes in the optic nerve and, consequently, visual field defects. Its occurrence is often directly associated with increased intraocular pressure (IOP), which is an important risk factor; however, it is insufficient as a diagnostic tool owing to the numerous patients with normal-tension glaucoma [2].

All types of glaucoma have progressive optic nerve damage in common. In most cases, visual loss occurs slowly, initially leading to mid-peripheral visual loss; in advanced stages, it affects the central vision leading to irreversible blindness.

The traditional basic diagnosis of glaucoma is made by an ophthalmologist based on the IOP data, a degree of functional impairment resulting from the disease through perimetry, and a manual evaluation of the optic nerve and retinal nerve fiber layer (RNFL) structures from fundus images, which are commonly obtained by indirect ophthalmoscopy with a conventional retina photo camera or slit lamp [3]. In high-income countries, usually several analyses with optical coherence tomography (OCT) of the optic nerve and RNFL are added, whose evaluations are represented by graphs, which also allow for comparisons with age-matched normative data [4].

The fundus examination is a non-invasive and vital test for detecting systemic diseases of the microcirculation in the human retina, such as glaucoma, which is confirmed by the presence of directly observable features in the optic disc [5]. This includes the whitish central part indicating the absence of neural tissue (called the “optic cup”), glaucomatous optic neuropathy, changes in the RNFL, and peripapillary atrophy (PPA). It is also evaluated via the cup-to-disc ratio (CDR), calculated based on the ratio of the vertical cup diameter (VCD) to the vertical disc diameter (VDD). The cup-to-disc ratio (CDR) is measured as a fractional percentage, and optical cups greater than 0.65 indicate possible abnormalities [6].

**Figure 1:**
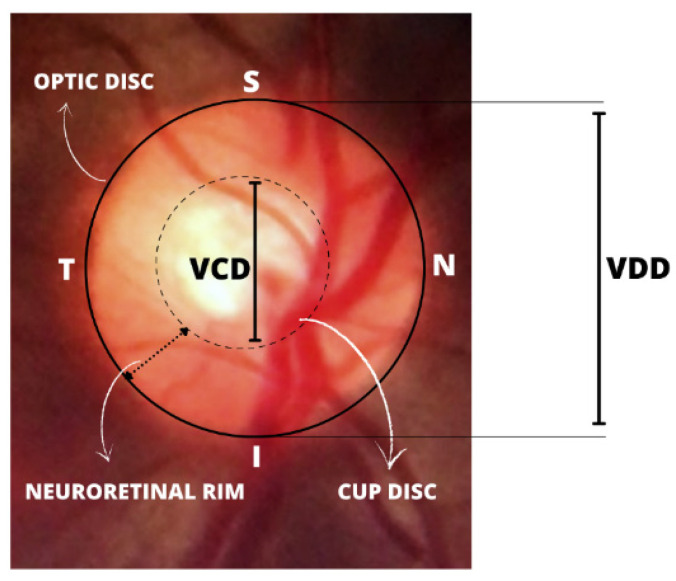
Illustration of the inferior, superior, nasal, temporal (ISNT) rule pattern and important structures for the diagnosis of glaucoma.

Further observations can be made about changes in the thickening of the neuroretinal rim (which follows a specific pattern of width in healthy people). In the neuroretinal rim, the inferior rim (I) is the widest, followed by the superior rim (S), nasal rim (N), and finally the temporal rim (T). This pattern collectively identified as the inferior, superior, nasal, temporal (ISNT) rule, exemplified in Figure 1, is widely used in optic nerve-head evaluation [7,8]. Additionally, the size of the optic disc is important; large discs usually have large cups (resembling and overestimating glaucoma), and in small discs, even a small cup might be glaucomatous (underestimating glaucoma).

Although glaucoma is incurable, proper treatment can retard its progression to more serious conditions. Therefore, an early diagnosis is important for glaucoma patients. In addition, scientific results from Europe demonstrated that resource utilization and direct medical costs of glaucoma management increase with worsening disease [9].

Population screening is a broader approach for the early detection of glaucoma and is a diagnostic method that can be applied to society as a whole or at least in high-risk groups. However, studies have shown that in countries such as the UK and Finland, population-based glaucoma screening by traditional diagnostic methods is not feasible owing to the high cost of implementation and maintenance, and the relatively low prevalence of the disease in the general population (approximately 3.5%) [10,11].

Despite the impracticality of population screening for glaucoma by conventional means, deep learning (DL), especially convolutional neural networks (CNNs), have been widely used in the field of medical images and are considered pattern recognition tools that can aid in diagnosis of eye diseases, suggesting, for example, different methodologies and approaches to detect diseases such as cataracts [12,13,14] and glaucoma [15,16], from digital images. The use of DL has also been demonstrated in studies of diabetic retinopathy diagnosis on a large scale. This evolution is because of several factors, such as the development of sophisticated algorithms and the availability of eye fundus image datasets for these studies.

**Figure 2:**
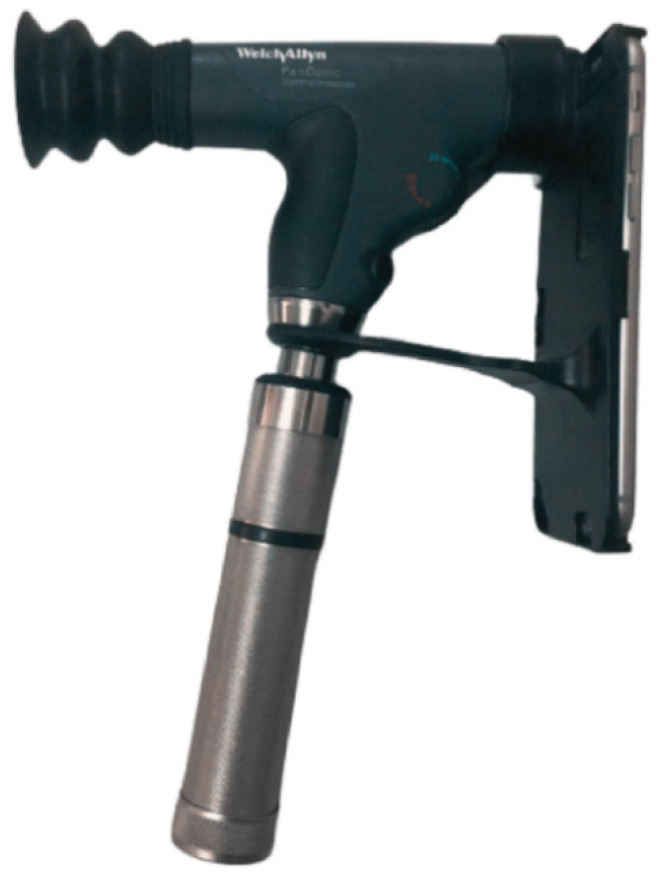
Panoptic ophthalmoscope Welch Allyn 11820.

With growing technological advances in both algorithms and physical media for ophthalmology, several portable ophthalmoscopes for smartphones have been developed and are sharing space with traditional ophthalmology cameras in the acquisition of fundus images [17,18,19]. The Panoptic Welch Allyn ophthalmoscope [18], shown in Figure 2, is a device that features easy image capturing, portability, easy data transfering, and compatibility with smartphones and data acquisition applications. Compared to conventional ophthalmic equipment, the device has a lower image resolution; however, owing to its general quality, it has great potential for telemedicine, patient screening, and clinical examinations, in addition to its low cost when compared to the traditional equipment.

The panoptic ophthalmoscope is already widely known and used by healthcare professionals, but in terms of machine learning (ML), it remains to be seen whether the algorithms currently studied, trained, and evaluated to automatically diagnose glaucoma based on fundus images obtained from conventional equipment will have similar accuracy when trained and evaluated with fundus images obtained from smartphones and the panoptic ophthalmoscope.

One difficulty in using artificial intelligence (AI) to test smartphone images for glaucoma detection is that there are no publicly available datasets for such studies, as all publicly available datasets are obtained from large conventional cameras. Therefore, given this deficiency and the ongoing advances in the smartphone-assisted imaging of the eye fundus, as well as the availability of DL algorithms for pattern recognition in digital images, the focus of this study is to build a new dataset containing images labeled for glaucoma acquired via a smartphone and the panoptic ophthalmoscope.

To enhance automated glaucoma diagnostic studies using smartphone images, a DL algorithm with a final hit rate of 90.0% was developed to classify the images in this new collection as having or not having glaucoma. This demonstrates that the integration of these new technologies can help under-resourced primary care centers and provide diagnostic support to ophthalmologists.

The remainder of this paper is organized as follows: Section 2 presents a literature review of related research. Section 3 presents the developed Brazil Glaucoma (BrG) dataset. Section 4 details the pre-trained models used in this study and analyzes the results obtained in the classification of glaucoma. Finally, Section 5 discusses the overall study, and Section 6 provides concluding remarks and outlines the scope for future work.

## 2. Related Work

This section presents relevant and recent work conducted for examining glaucoma diagnosis using DL. First, the main datasets containing marked glaucoma images are introduced and followed by related work on classifying glaucoma from fundus images.

### 2.1. Public Glaucoma Datasets

Listed below are major image sets that have been publicly found on the Internet and have been used by various glaucoma-classification algorithms.

ACRIMA: Created by the Spanish Ministerial Organization for Economy and Competition. The dataset consists of 396 glaucoma images and 309 normal images, for a total of 705 images acquired with a Topcon TRC retinal camera configured for a 35° field of view. Two glaucoma specialists labeled all the images of this dataset [20].

DRIONs: The images were acquired from Hospital Miguel Servet, at the ophthalmology service in Zaragoza, Spain. The dataset comprises 110 fundus images (55 healthy and 55 glaucomatous). All images were obtained from Caucasian subjects using a conventional color analog fundus camera centered on the optic disc region and stored in slide format. Subsequently, the images were scanned using a high-resolution HP-PhotoSmart-S20 scanner and saved at a size of 600 × 400 pixels [21].

DRISHTI: The dataset contains 101 fundus images (31 healthy images and 70 glaucoma images) acquired at the Aravind Eye Hospital in Madurai, India. The images were captured using a traditional high-resolution OD-centric camera with 30° of view and a size of 2896 × 1944 pixels [22].

DRIVE: These fundus images were obtained for extracting vessels for an eye screening research project in the Netherlands. The database includes 40 fundus images (34 healthy and 6 glaucoma) annotated by two ophthalmologists. The images were taken with a Canon CR5 3CCD non-mydriatic camera with a field of view of 45° and dimensions of 565 × 584 pixels [23].

GLAUCOMADB: No localization record was obtained for the dataset. It consists of 120 fundus images (85 healthy images and 35 glaucomatous images) from a larger set of 462 images. The glaucoma labels were applied by two ophthalmologists. The images were taken with a TopCon TRC 50EX camera with a resolution of 11504 × 1000 pixels [24].

HRF: The images were collected at a single clinic in the Czech Republic. Out of a total of 45 images, 15 are glaucomatous, 15 normal, and 15 are labeled as diabetic retinopathy. All fundus images were acquired with a Canon CR-1 mydriatic camera and different acquisition settings with a 45° field of view and 3504 × 2336 pixels. There are no records of how many ophthalmologists were used to label the images [25].

MESSIDOR: The images were acquired by three ophthalmological departments in France. The dataset contains a total of 1200 images of different diseases, but only 100 images are labeled for glaucoma (28 with glaucoma and 72 normal). The images were acquired using a Topcon TRC NW6 non-mydriatic camera with a 45° field of view and sizes of 1440 × 960, 2240 × 1488 or 2304 × 1536 pixels [26].

ORIGA: This dataset has a total of 650 fundus images divided into 168 glaucoma images and 482 normal images. It was constructed using retinal imaging data collected from the Singapore Malay eye study in conjunction with the Singapore Eye Institute. Disc-related statistics (such as ISNT compliant CDR and RNFL defects) and manually segmented results for optical discs and optical cups are provided for each image [27].

PAPILA: These images were acquired by ophthalmologists or technicians at the Unit of the Reina Sofía University Hospital, Spain, using a Topcon TRC NW400 retinal camera non-mydriatic with a resolution of 2576 × 1934 pixels. The PAPILA dataset, which involved 244 patients, provides a total of 488 images divided into 333 healthy and 155 with glaucoma or suspected glaucoma. Labeling for glaucoma was based on clinical data [28].

REFUGE: This dataset contains 1200 images divided into 120 images from glaucoma patients and 1080 from healthy patients. Image acquisition was performed using two retinal cameras (a Zeiss Visucam 500 fundus camera and a Canon CR-2 camera with resolutions of 2124 × 2056 and 1634 × 1634 pixels, respectively). This dataset also provides information on the optic disc and optic cup prepared by seven glaucoma specialists at the Sun Yat-Sen Eye Center, (located in Guangzhou, Guangdong Province, China) [29].

RIM-ONE DL: The RIM-ONE-DL dataset [30] was created in 2020 by combining three open versions of the eye fundus image set called the retinal image database for optic nerve evaluation (RIM-ONE). The first open version of the RIM-ONE eye fundus image set was published in 2011 by (Fumero et al.) [31]. The second version published in 2014 was designed as an extension of the first; subsequently in 2015 (Fumero et al.) [32] published the third version of the dataset. They are referred to in this paper as RIM-ONE v1, v2, and v3, respectively. The final RIM-ONE-DL dataset consists of 313 images of healthy subjects and 172 images of glaucoma patients. Because the dataset is newly created, most previous academic studies are based on either RIM-ONE v1, v2, or v3 images. Therefore, they are described below.

- RIM-ONE v1. The main objective of this study in 2011 was to provide a database of retinographies of 118 healthy subjects and 51 patients classified in various stages of glaucoma. Fundus images were acquired using a non-mydriatic Nidek AFC-210 camera with a Canon EOS 5D Mark II body with a field of view of 45°.
- RIM-ONE v2. It contains 255 images of healthy individuals and 200 images of patients with glaucoma. It is an extension of the first version and presents images manually segmented by a specialist doctor. Images were taken at HUC and HUMS using the same camera as in version 1.
- RIM-ONE v3. It contains 85 images of healthy individuals and 74 images of patients with glaucoma. Images were captured only at the HUC with a non-mydriatic Kowa WX 3D fundus camera with a full resolution of 2144 × 1424 pixels.
- G1020: G1020 images were collected at a private clinic in Kaiserslautern, Germany, between 2005 and 2017. Images were acquired with a 45° field of view using mydriasis. The dataset contains 1020 publicly available fundus images (724 healthy and 296 with glaucoma). Labeling of the images is provided, as well as segmentation of the optic disc and optic cup. In the final dataset, the images have sizes between 1944 × 2108 and 2426 × 3007 pixels [33].

### 2.2. Glaucoma Classification Algorithms in Fundus Images

In previous work seeking glaucoma pattern recognition in fundus images, some researchers [34,35,36] focused on implementing algorithms for segmenting and measuring the CDR or applied it to algorithms in the analysis of the texture of fundus images. The objective of the final classification was reached with the help of various architectures such as *k*-nearest neighbors (KNN), support vector machine (SVM), decision trees, and naïve Bayes.

Shinde [34] used a computer-aided diagnosis system. The optic disc region was segmented from the optical cup with the aid of a U-Net architecture, attribute extraction was applied from the segmented region, and then glaucoma classification was performed using SVM. Sreng et al. [35] introduced an automatic two-stage glaucoma screening system. The system first segmented the disk region for classification and then the authors used pre-trained CNN architectures for three purposes: transfer learning, learning the feature descriptors using a support vector machine and finally again with both methods.

Abdel-Hamid [36], proposed a new generic wavelet using a glaucoma detection algorithm that has the advantage of being applied on both the time and frequency. This study used two public image sets for the algorithm performance analysis (GLAUCOMADB and HRF). An accuracy of 96.7% and area under the receiver operating curve (AUC) of 94.7% were achieved for the HRF dataset using the KNN algorithm. Singh et al. [37] used various statistical features of fundus images and ISNT and CDR rate measurements. The output of the proposed model was obtained through an ensemble, which is the concatenation of the outputs of the individual classifiers. To build the ensemble, they used four algorithms (SVM, KNN, naïve Bayes, and artificial neural network (ANN)) and achieved 98.60% accuracy in classifying glaucoma.

In other studies [38,39,40,41,42], researchers used CNNs for classifying eye fundus images because of their obvious advantages in image processing. CNNs are DL algorithms whose architecture resembles a multilayer perceptron ANN, usually with more layers and convolutional operations in at least one of them [43]. One of the difficulties is that these algorithms need to be trained on large datasets that are not always publicly available. Thus, using private image sets, Li et al. [44] proposed an attention-based CNN for glaucoma detection. The algorithm showed an accuracy of 95.3%. They used a dataset comprising 11,760 fundus images, with 4878 labeled glaucomatous and 6882 normal.

Moreover, using private image sets, Ting et al. [45] trained a DL-based algorithm with 125,189 fundus images to detect possible glaucoma. The DL performance was evaluated on 71,896 test images with an AUC of 0.942% in classifying possible glaucoma, demonstrating the diagnostic and computational power of ML algorithms. Continuing with the use of private datasets, Li et al. [46] developed a DL algorithm to detect glaucomatous based on 48,116 fundus photographs. The effectiveness of the algorithm was measured from 8000 validation images, which yielded an AUC of 0.986.

Liu et al. [47] used public and private datasets to classify glaucoma and concluded that a deep learning-based algorithm can identify glaucoma from monovision fundus images with high accuracy. Chen et al. [48] proposed a DL algorithm by means of an ensemble that integrates four depth streams at different levels of an eye fundus image, as outputs, and combines all the outputs of these depth streams to obtain the final classifier result. Experiments on two datasets were shown to be efficient in classifying glaucoma.

Regarding the insufficient publicly available images and the need for a large number of images to train the CNN architecture, among the possible artificial data augmentation techniques, there is also the possibility of using transfer learning; that is, reusing a pre-trained model on another larger dataset to solve a new problem, for example, the large ImageNet dataset [49]. Pre-trained networks have been used for glaucoma detection from fundus images in public and private datasets. For example, Diaz-Pinto [20] used five different pre-trained architectures as glaucoma classifiers using only publicly available datasets, and their best architecture showed a mean area under the ROC curve of 0.96 for glaucoma classification.

Algorithms with transfer learning were also evaluated in several other studies as in [44], and also available at [50,51], where private datasets were used and the obtained accuracy exceeded 90%. Christopher et al. [52] studied three different DL architectures. For each architecture, two different versions were evaluated: native learning and transfer learning. In all cases, the authors showed that transfer learning can improve performance and reduce the training time of the algorithms. Although the reported works and datasets present a diversity of fundus images and diverse ethnicities because they were built in different locations, all the sets in question were obtained using conventional fundus cameras, and no set of images with labeling for glaucoma has so far been obtained with the aid of smartphones and made publicly available.

## 3. Dataset Brazil Glaucoma (BrG)

This section first presents the panoptic ophthalmoscope and smartphone used in the fundus image acquisition, the ocular images acquisition site, and finally, the cropping of the images and preparation for the glaucoma classification algorithm.

The device used for the fundus examination was the Welch Allyn 11820 Panoptic ophthalmoscope [18], identical to the model shown in Figure 2.

The iExaminer application transforms the panoptic ophthalmoscope into a mobile digital imaging device, which allowed users to view and take photographs of the fundus of the eye through a smartphone. Its optical design produced its own light and provided easy access to small pupils with good background lighting, allowing photography without pupil dilation. To take the photographs, the ophthalmoscope was powered by battery (an original 3.5 volt), providing a field of view up to 25° with focus adjustment from −20 to +20 diopters [18]. The smartphone used in the study was an Apple iPhone 6s device with a 12-megapixel camera.

### 3.1. Image Acquisition Process 

**Figure 3:**
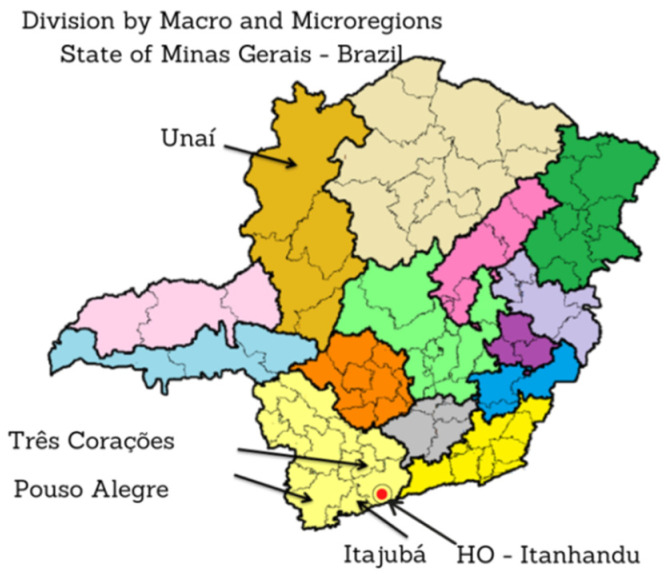
Map of Minas Gerais (MG) indicating the cities where the fundus photographs were taken and the region where Hospital de Olhos (HO) is located.

The fundus images of the dataset established in this study were obtained from two different locations, namely, the Hospital de Olhos (HO), do Sul de Minas Gerais (MG), and Policlínica de Unai MG between the months of April 2021 and February 2022, as shown in Figure 3.

Glaucoma images were collected from Brazilian patients treated at the HO by southern MG [53], with headquarters in the city of Itanhandu, Brazil. This is a private hospital with a glaucoma treatment program that covers an area of approximately 2 million inhabitants. The hospital maintains an agreement with the Unified Health System (SUS) in Brazil [54], which is responsible for funding service providers such as the HO and public health centers according to the guidelines of the Ministry of Health [55]. The HO offers treatment to patients who have had their glaucoma diagnosis confirmed in other regional health clinics, or to those patients who are diagnosed through the screening quotas offered by the HO.

Images of patients without glaucoma were collected during elective ophthalmology consultations at the Polyclinic Health Center in the city of Unai, MG, Brazil. The clinic operates in cooperation with SUS and offers medical and ophthalmic care to the general population.

According to the legal obligations, all patients seen at the HO were required to undergo the following exams every three months: anamnesis; measurement of visual acuity; IOP measurement; campimetry; ultrasonic pachymetry exam that evaluates central corneal thickness, which can influence the IOP estimation; and optic nerve evaluation using a slit lamp. The HO welcomes patients who presented themselves with at least two of the following diagnoses: mean untreated IOP above 21 mmHg, typical optic nerve damage with neuroretinal rim loss identified by fundus biomicroscopy with (CDR at or above 0.5), or visual field compatible with optic nerve damage. Thus, images with glaucoma were labeled based on clinical findings during consultations and examinations offered by the HO.

The collection methodology also considered the acquisition of images of patients without glaucoma. The difference between the treatment program offered by the HO and the consultation program offered by the Unai Polyclinic is the intended objective. However, as the goal at the Unai Polyclinic is to provide more general elective consultations, the exams included only refraction, IOP measurement, visual acuity, and fundus examination with a slit lamp. The absolute truth of each label was confirmed directly by ophthalmologists in charge of local consultations. In this way, the absolute truth for each image labeled as glaucoma-free was confirmed by the ophthalmologists responsible for the local consultations.

In this study, 1000 volunteers had their eye fundus photographed. The volunteers were divided into 500 patients with glaucoma (treated at the national glaucoma program) and 500 patients without glaucoma (who had their eyes examined at the municipal polyclinic in Unai/MG). All volunteers had both eyes (left and right) photographed. Thus, a total of 2000 fundus images were taken.

For both glaucoma and non-glaucomatous patients, those between the ages of 18 and 80 years were selected, with approximately an equal number of men and women. Patients who voluntarily consented to participate in the study had their eyes photographed by a non-medical professional using a smartphone with the panoptic ophthalmoscope while waiting for eye care.

A relevant feature presented in the images of the BrG dataset is that the images were not divided considering the stages of glaucoma. However, it is possible that there is a balance in the database between the stages i.e., (i) early, (ii) intermediate, and (iii) advanced stages of the disease which is due to the population campaigns proposed by HO to combat the disease, in which people are motivated and educated to seek the ophthalmologist more often, enhancing early disease diagnoses. Therefore, the BrG database is composed of images of patients who sought OH out of necessity; that is, they already had structural and functional damage that compromised their vision and thus sought ophthalmic care.

Other patients sought care because of the greater availability of consultations for the regional population. Many patients sought care in the HO, attracted by campaigns to combat glaucoma, and had the glaucoma diagnosis early, i.e., before functional damage compromised their quality of life. Moreover, considering the time of implementation of the glaucoma consultation and treatment program by the HO and the impact and stability of the discovery of new cases of the disease in the southern region of MG, it is possible to infer that the BrG dataset was constituted with a more uniform distribution among the stages of the disease.

### 3.2. Preprocessing of the Eye Fundus Images

**Figure 4:**
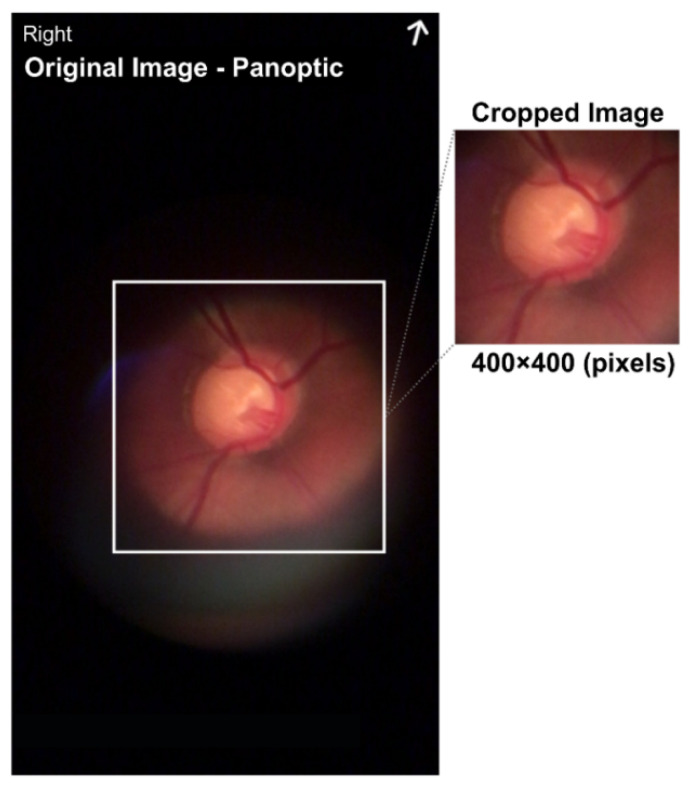
Image size of 720 × 1280 pixels with a center cut of 400 × 400 pixels in the region representing the optical disk.

To take the pictures, a short clip was recorded; then, the five best images were manually pre-selected based on optimal focus and visualization of the vasculature, and finally the best among the five images was selected manually. The images were acquired using the red, green, and blue (RGB) color representation and the joint photographic experts group (JPEG) format. All images were taken with the eyes undilated using the ophthalmoscope centered on the optic disc with a field of view of approximately 25°. Poor-quality images in terms of positioning of the optic disc region and of low-contrast were discarded. To build the dataset, the optical disk region was extracted from the original image by eliminating the surrounding black region, thereby obtaining an image of approximately 400 × 400 pixels, as shown in Figure 4.

**Figure 5:**
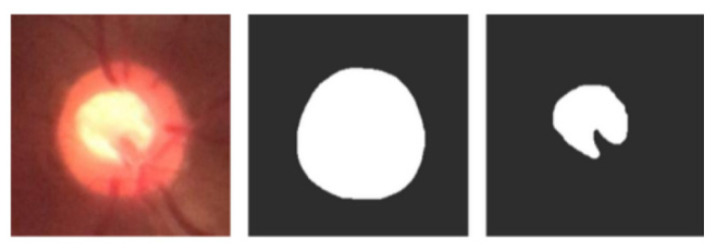
Example of optic disc and optic cup masks used in segmentation algorithms.

The images were cropped in the center and saved in portable network graphics (PNG) format. The cropping of the images was performed semi-automatically using the bounding box tool. The cropping corresponded to a rectangular area superimposed to focus on the optical disk. The images were not processed further. The new public dataset was called Brazil Glaucoma (BrG). All images were anonymized of personal data and for every image in the dataset, there is an optic disc mask and an optic cup mask that can be used by segmentation algorithms, as shown in Figure 5. The masks were created using the Easy Paint Tool SAI 2.

**Figure 6:**
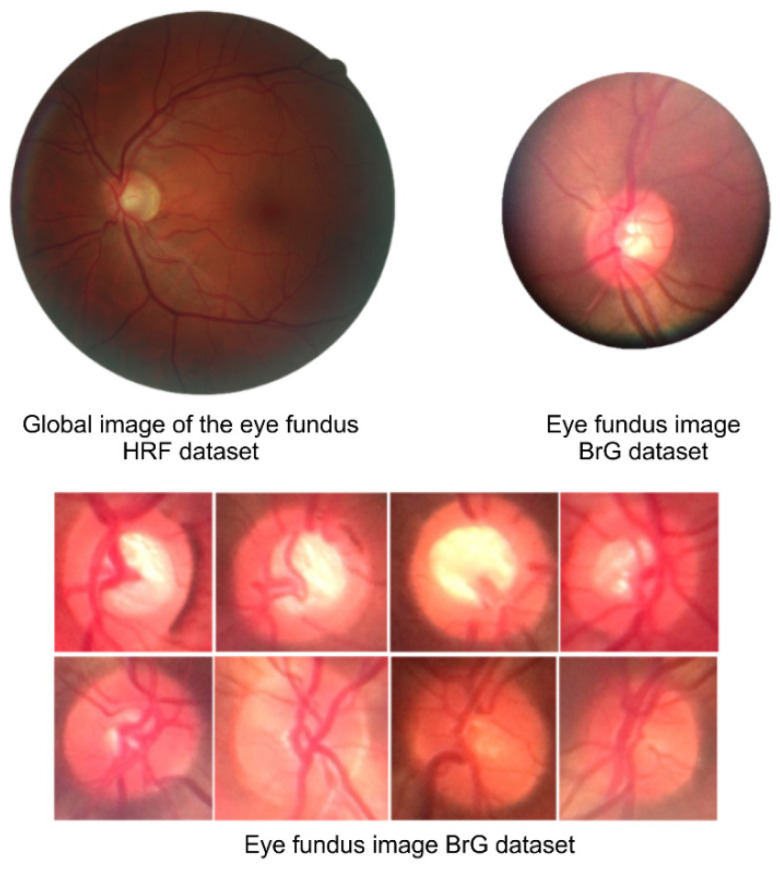
Fundus image comparison shows differences between global images and portable ophthalmoscope images that comprise the BrG dataset.

Figure 6 compares the global image with the fundus image, i.e., it shows the entire fundus of the eye and the image from the smartphone-attached panoptic ophthalmoscope, which shows images centered on the optic disc region.

### 3.3. Images with Noise

During the acquisition of the BrG dataset, we found that there are three potential types of noise that can interfere with the overall accuracy of the DL algorithms. The first type, or just a characteristic, is related to the low-contrast and the appearance of some images darker than others. This effect can occur as a result of the power supplied to the device, which was via a rechargeable 3.5-volt battery. Therefore, when working continuously, the first images may appear with higher lighting, whereas subsequent images may appear with lower lighting. Although panoptic devices have lighting adjustments, controlling these effects is difficult.

The second type of noise arises from external lighting. This noise occurs when ambient lighting cannot be controlled. To reduce these effects, the ophthalmoscope has an eye shield that blocks external light and improves the contrast of the image. However, depending on the position of the face or the physiognomy of some people, this shield may allow the passage of external light, which can cause unwelcome noise.

The third, and most frequent, noise type is obtained with the light of the device itself. Specifically when pointed at an improper angle, the device can cause reflections that can be harmful to the final images.

**Figure 7:**
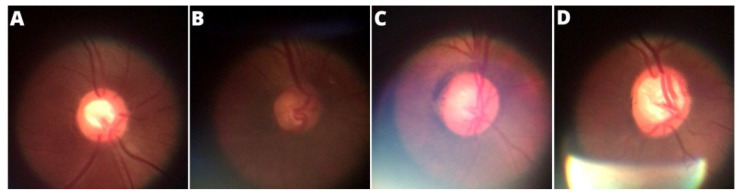
Main noise types presented in the panoptic ophthalmoscope images: (**A**) ideal image, (**B**) low lighting, (**C**) external noise, (**D**) light focus noise.

Figure 7 shows examples of an ideal image, an image with noise caused by insufficient lighting, an image with noise caused by external light interference, and finally an image with noise caused by the ophthalmoscope’s own light due to the often inadequate adjustment to take the photo. However, as already mentioned, images with compromising qualities were discarded and not counted in the formation of the BrG dataset.

## 4. Model Selection and Training

The objective of this image classification stage is to classify an image input to a DL algorithm into two categories: glaucoma or glaucoma-free. To apply these image classifications, we divide the process into three steps, namely: the selection of CNN models, experimental evaluation and ensemble construction and results.

### 4.1. Selection of CNN Models

The DL algorithms applied in this research were CNN models pre-trained on the ImageNet dataset [49] that allowed transfer learning. Table 1 presents the seven CNN models selected in this study. The classifiers were chosen because they are widely used pattern recognition models for digital images, provided by the Keras library [56].

To improve the overall accuracy of the final classification of images, the outputs of the CNN models presented in Table 1 were concatenated to form an ensemble model that combined the decisions of the individual classifiers to classify the test images. To build the ensemble model, we first trained each individual classifier. To apply training, the BrG dataset was divided into 70% for training and 30% for testing. The division was performed at the patient level, which means that all images of a patient were included in the same part of the dataset (training or testing). To use the hyperparameter comparison of the DL models, we split 20% of the images from the training set to create a validation set.

**Figure 8:**
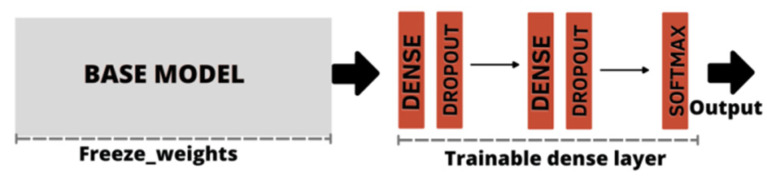
Freeze weights base model, followed by dense layer construction with dropout application. The output was obtained by the softmax activation function.

As the CNN classifiers were configurable, before training, we adjusted the parameters for application on the BrG dataset. Thus for each of the CNN models listed in Table 1, through a process called weight freezing, we froze part of the model and kept the weights and information learned in pre-training on the ImageNet dataset. We then added two new trainable layers on top of the frozen layers, and finally trained these new layers using the training images from the new BrG dataset as input, as shown in Figure 8.

For backpropagation applications, the adaptive moment estimation (Adam) optimizer was used as the loss function in the classifier [64]. To prevent the network from losing generality (a phenomenon known as overfitting), a technique called early stopping was applied; that is, we attempted to stop training the algorithm at the optimal learning point.

Data augmentation was also applied to artificially generate new samples of training data to increase the generality of the model. In this study, image rotation, scaling, and translation were applied. A dropout rate of 0.2 was used for fully connected layers to overcome overfitting.

The output of the CNN models shown in Table 2 was configured with an activation function (softmax) such that the network accepts a digital image as input and generates the probability that the input image represents a patient with or without glaucoma as output.

### 4.2. Experimental Evaluation

**Figure 9:**
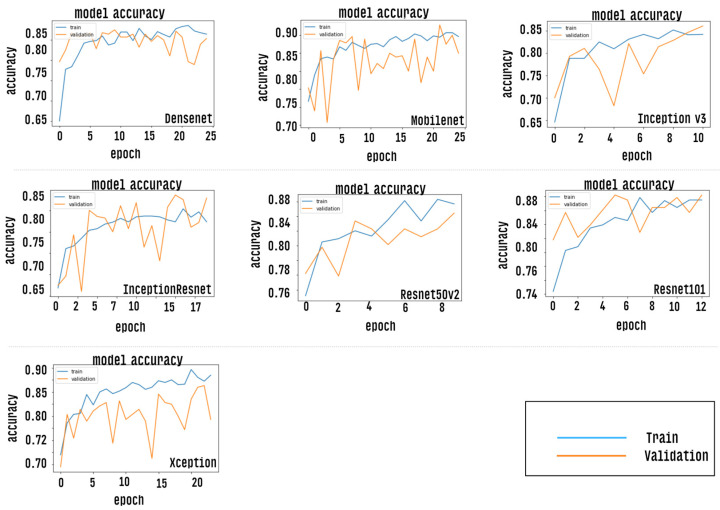
Shows an accuracy curve indicating the performance for each trained CNN model.

**Figure 10:**
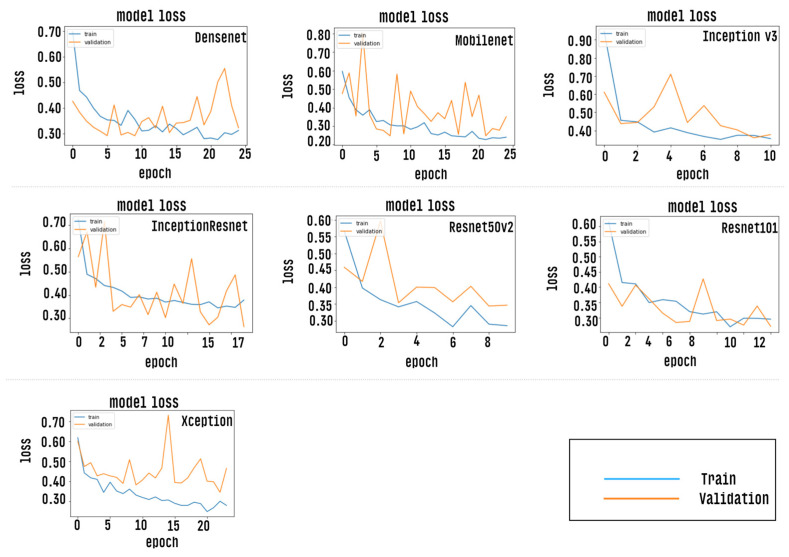
Shows a loss curve indicating minimum loss for each of the individual CNN models trained.

After the training stage, the accuracy of each CNN model is measured by passing the test dataset as input, however, prior to this measurement, the CNN models were evaluated via the accuracy curve and loss curve parameters. This evaluation was performed by passing the validation set as input of classifiers. The results of this step can be verified as shown in Figure 9 and Figure 10. Results correspond to CNN models trained with a defined number of epochs using a technique called early stopping. The graph shows values close to the overall mean for the five-fold cross-validation.

After the training and validation phases, all CNN models are tested using the test set as input and the global accuracy calculations for the proposed set were calculated using the following statistical equations.

Accuracy (AC) = (TP + TN)/(TP + FN + TN + FP)(1)

Sensitivity (SE) = TP/(TP + FN)(2)

Specificity (SP) = TN/(TN + FP)(3)

Precision (Pr) = TP/(TP + FP)(4)

F-Score (F1) = 2TP/(2TP + FP + FN)(5)

To calculate the accuracy, it is denoted that: TP characterizes the true positive results, TN explains the true negative ones, and false positive (FP) and false negative (FN) denotes the incorrectly identified classes [65]. The F1 score can be interpreted as the harmonic mean of precision, where the best value of the F1 score is 1 and the worst value is 0. The relative contributions of the metric Kappa (K), are analyzed in the same way as the F1 metric is analyzed. The Kappa coefficient is a statistical method used to assess the level of agreement or reproducibility between two sets of data [66].

In the analysis, the individual classifiers classified the images of dataset BrG into ‘positive’ or ‘negative’ glaucoma, as shown in Table 2. The accuracy corresponded to the average of the results obtained by five-fold cross-validation.

**Figure 11:**
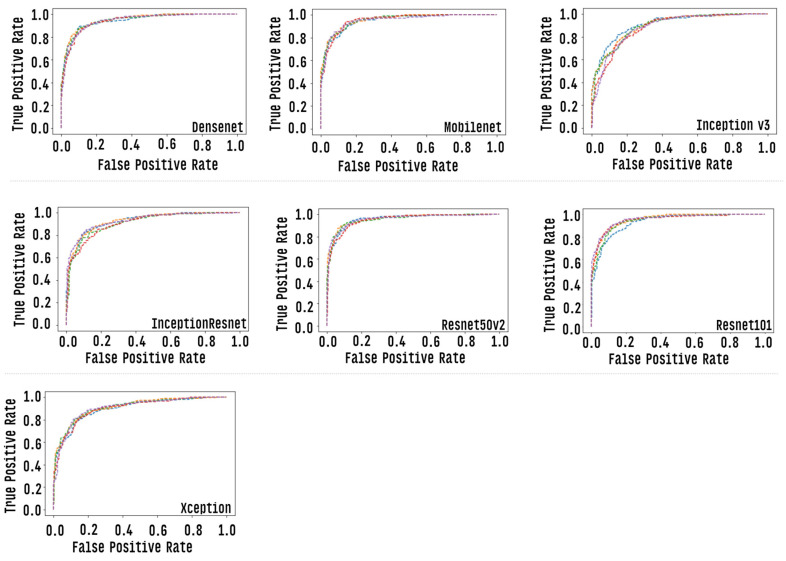
Roc curve of each of the individual CNN models, each row corresponds to one round of cross-validation.

We graphically evaluate the results of individual CNN models via the “area under the ROC curve (AUC)”, which corresponds to a graph showing the performance of a model across all classification thresholds. This curve plots two parameters: true positive rate and false positive rate. Figure 11 presents the ROC curve of each classification model. AUC values range from 0.0 to 1.0, with a threshold between classes of 0.5, so a model that predicts 100% correct has AUC equal to 1 [67].

**Figure 12:**
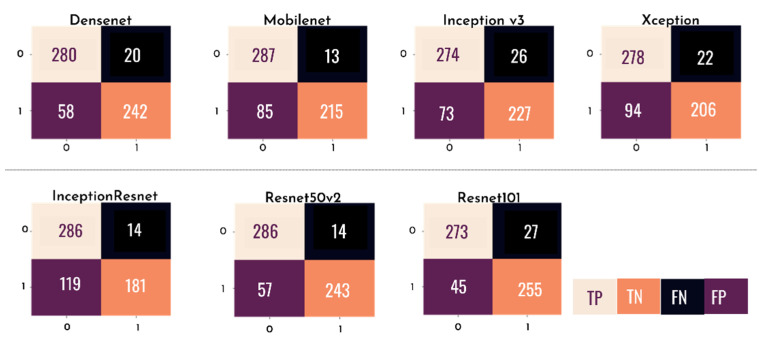
Confusion matrix of each of the individual CNN models.

Figure 12 presents a confusion matrix for each of the individual models tested, where the rows represent the predicted values of the model and the columns represent the actual values. With this matrix, it is possible to analyze, through sensitivity, the probability of a clinical case of glaucoma being correctly diagnosed by the test and, through specificity, the probability of a non-clinical case being correctly identified.

### 4.3. Ensemble Construction and Results

**Figure 13:**
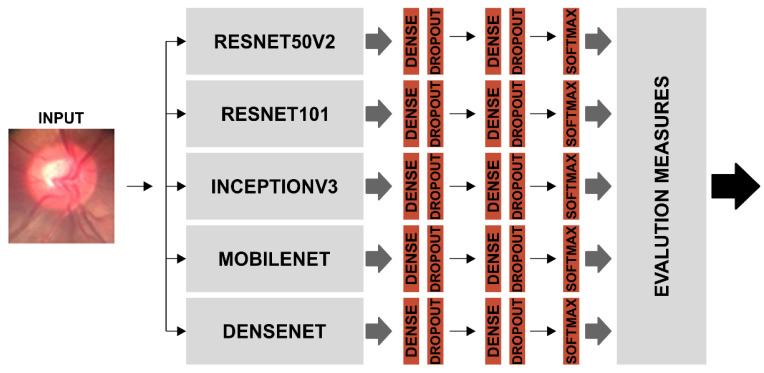
Ensemble model using the individual classifiers that was most accurate in classifying BrG dataset images.

The individual accuracy values of the Resnet50v2 and Resnet101 algorithms obtained the best results for the overall classification of the eye fundus images in the BrG dataset; however, seeking to further improve the accuracy of the overall classification of the eye fundus images under study, we grouped the individual classifiers into an ensemble, as shown in Figure 13.

There are several approaches to the combinatorial programming of classifiers, in this work; the ensemble results were obtained by averaging the probabilities of the individual classifiers to acquire the unique probability that an image represented either a patient with glaucoma or a non-glaucoma patient.

To select the best combination of classifiers to form the ensemble, combinations of the seven algorithms listed in Table 2 were tested, excluding the least accurate algorithm at each combination tested: Combination 1 was conducted by concatenating the outputs of all seven individual classifiers, and Combination 2 was conducted by concatenating the outputs of the six most accurate individual models. These combinations were performed until Combination 6, which had only the two individual algorithms with the highest accuracy, as shown in Table 3.

Table 4 lists the ensemble results for all combinations established by the method used.

Finally, after considering the highest accuracy value, the best ensemble was seen as that formed by Combination 3, with the addition of classifiers Resnet50v2, Mobilenet, Densenet, InceptionV3, and Resnet101, and thus consolidated the final Ensemble with the best performance in the classification of BrG images, as shown in Figure 13.

**Figure 14:**
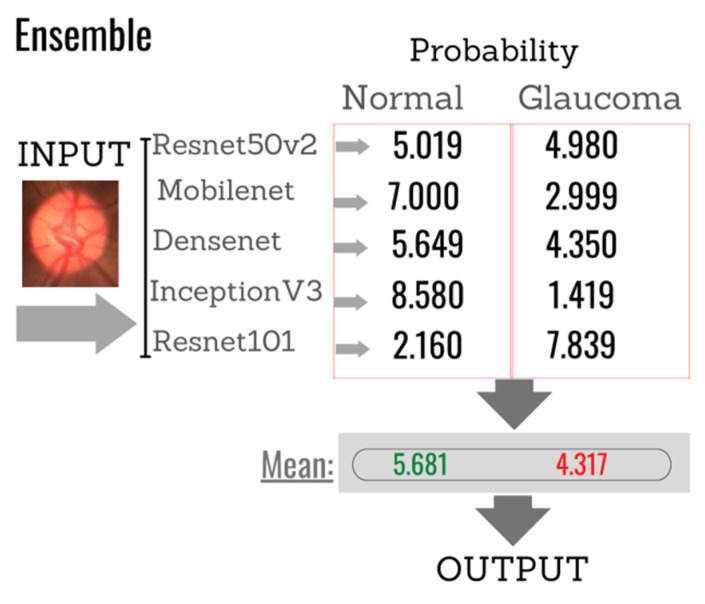
Example of the final response of the classifier based on the individual responses of each of the algorithms selected to compose the Ensemble.

For a better understanding of the combination of individual classifiers and formation of the ensemble, Figure 14 shows an example in which the images must be classified into two categories: (normal or glaucoma). Assuming that the softmax function is used in the output layer of each CNN classifier, the test output is the probability that the input image belongs to one of the given classes. Thus, the final Ensemble response is derived from the average of these probabilities, generating a single probability of whether or not an image is glaucomatous. In the illustration given as an example, the final result shows that the image has a 5.649% probability of not being glaucoma and a 4.350% probability of being glaucoma. Therefore, based on this example, the image would be classified as non-glaucoma.

**Figure 15:**
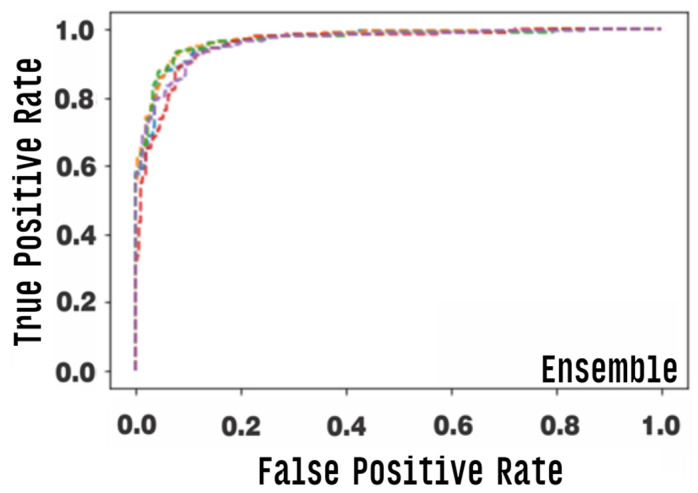
Area under the ROC curve (AUC) for the ensemble model, each row corresponds to one round of cross-validation.

**Figure 16:**
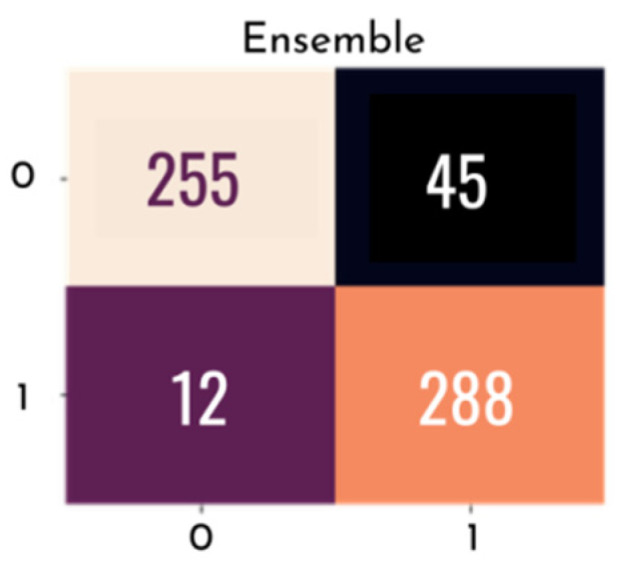
Confusion matrix with final ensemble result.

Figure 15 and Figure 16 graphically show the integrated ROC curve and the confusion matrix, obtained using Ensemble. For the presented results, the mean of the five-fold cross-validation was considered.

The best combination of the ensemble exhibited an accuracy of 0.905, and a final AUC of 0.965%, with a confidence interval of 0.950–0.965%, a final sensitivity of 0.850, and a specificity of 0.960. Other metrics used are listed in Table 4.

## 5. Discussion

First, considering the new BrG dataset and comparing it with the related datasets, we observed their characteristics. The main difference between BrG and other datasets is the acquisition method, of which only the BrG database is composed entirely of images obtained by connecting a smartphone with a direct handheld ophthalmoscope, which is less expensive than the acquisition methods of the other datasets in evidence.

Second, BrG images have a smaller field of view and resolution than those of other related datasets. In this sense, all datasets presented global images, i.e., covered the entire area of the eye fundus, except for the BrG images that focused only on the area of the optic disc owing to the limitations of the light range to allow global images.

The fact that BrG does not present global images might be a disadvantage in some cases where this image type is necessary; however, considering that the disease under study is glaucoma, this particularity might not represent a problem, as the area of the optic disc represented the most important content in the diagnosis of glaucoma. However, all related work reported here only used the features observable in the optic disc region. Furthermore, Fu et al. [48] compared the accuracy of their algorithm taking global images and segmented images in the optic disc region. In all cases, the best accuracy was obtained using only the optic disc area, reinforcing that BrG images can be useful for the diagnosis of diseases harmful to the optic disc, such as the case of glaucoma. As for the resolution of the images, more tests are needed, especially tests focused on segmenting the structures of the optical disk because segmentation depends on sharper images.

Considering the number of images marked for glaucoma, the new BrG database outperformed publicly available datasets. As for the limitations, the BrG dataset was composed entirely using a single camera (smartphone), whereas, sets such as REFUGE and RIM-ONE were composed using multiple cameras.

Regarding the classification of glaucoma using an ensemble of CNNs, the 90.0% accuracy of the classification algorithm in the BrG dataset is consistent with the results obtained by other researchers, as one should not consider only the final accuracy result but the entire methodological process, from the acquisition of images to the classification results.

Therefore, analyzing the results of Diaz et al. [20], who also worked with several classifiers, a similarity can be noted between the final accuracy they obtained using high-resolution images and the accuracy achieved in this work. However, considering that the BrG dataset was built using low-resolution images, the results presented here are in accordance with the expectations of the classification algorithm. Furthermore, the performance of this algorithm can be improved by refining the parameters and applying more rigor to the acquisition of smartphone images; for example, by better controlling the environment in which the photographs are taken and the lighting offered by portable ophthalmoscopes. Such care can lead to the composition of a more homogeneous dataset, and factors such as these can improve the quality of images, providing greater final classification accuracy by DL algorithms.

## 6. Conclusions

In this study, a new dataset called BrG was built with images labeled and prepared for use by glaucoma-classification algorithms. Then, the accuracy of the classification of these images into glaucoma and non-glaucoma groups was analyzed with a combination of DL methods based on CNNs pre-trained for automatic glaucoma detection. As for the classification of glaucoma using an ensemble of CNNs, the 90.0% accuracy of the classification algorithm on the BrG dataset is consistent with the results obtained by other authors. It also shows that it is possible to use smartphone images for the classification of glaucoma through ML and was considered as a path to be explored by DL algorithms. Clearly, the study results showed that new portable technologies for fundus photography can be combined with AI algorithms and achieve satisfactory results in the overall accuracy of glaucoma detection tests. These technologies could enable screening projects for the disease, but there is a need for tests with a larger number of images and more refined classification algorithms. In future work, the BrG images will be tested in algorithms for segmentation of optic disc structures and applied in longitudinal work, as we seek to understand and map the evolution of glaucoma using AI algorithms.

## Figures and Tables

**Table 1:** Pre-trained CNNs with RGB color pattern used in this study.

CNN	Input Size
Densenet [57]	224 × 224 × 3–(RGB)
Mobilenet [58]	224 × 224 × 3–(RGB)
InceptionV3 [59]	299 × 299 × 3–(RGB)
InceptionResnet [60]	299 × 299 × 3–(RGB)
Resnet50v2 [61]	224 × 224 × 3–(RGB)
Resnet101 [62]	224 × 224 × 3–(RGB)
Xception [63]	299 × 299 × 3–(RGB)

**Table 2:** Results of individual classifiers.

CNN-Individuals	AC	SE	SP	Pr	F1	AUC	K
Densenet	0.870	0.933	0.807	0.828	0.877	0.954	0.740
Mobilenet	0.836	0.957	0.718	0.771	0.854	0.947	0.676
Inception-v3	0.835	0.913	0.757	0.789	0.847	0.930	0.670
InceptionResnet	0.778	0.953	0.600	0.706	0.811	0.932	0.557
Resnet50v2	0.881	0.953	0.810	0.833	0.889	0.956	0.763
Resnet101	0.880	0.910	0.850	0.858	0.883	0.949	0.760
Xception	0.806	0.926	0.686	0.747	0.827	0.919	0.613

**Table 3:** Combinations of CNNs evaluated in the ensemble construction.

CNN	Combinations					
	1	2	3	4	5	6
Resnet50v2	✓	✓	✓	✓	✓	✓
Mobilenet	✓	✓	✓	✓	✓	✓
Densenet	✓	✓	✓	✓	✓	
InceptionV3	✓	✓	✓	✓		
Resnet101	✓	✓	✓			
Inc-Resnet	✓	✓				
Xception	✓					

**Table 4:** Results obtained by combining the classifiers.

Combinations	AC	SE	SP	Pr	F1	AUC	K
1	0.861	0.767	0.957	0.946	0.847	0.967	0.723
2	0.865	0.763	0.966	0.858	0.849	0.968	0.730
3	0.905	0.850	0.960	0.955	0.899	0.965	0.810
4	0.865	0.770	0.960	0.950	0.850	0.964	0.730
5	0.838	0.703	0.973	0.963	0.813	0.963	0.677
6	0.853	0.730	0.976	0.969	0.832	0.961	0.700

## Data Availability

The BrG dataset presented in this study is openly available at: https://globaleyeh.com/ (accessed on 17 November 2022).
